# Upfront surgery, neoadjuvant chemoradiotherapy, or neoadjuvant chemotherapy for rectal cancer with lateral lymph node metastasis: A multicenter MRI and lateral lymph node dissection study

**DOI:** 10.1002/ags3.12873

**Published:** 2024-10-16

**Authors:** Takuya Miura, Kazushige Kawai, Hiromasa Fujita, Shinsuke Kazama, Hideki Ueno, Yusuke Kinugasa, Kazuhiro Sakamoto, Hirotoshi Kobayashi, Kenichi Hakamada, Yoichi Ajioka

**Affiliations:** ^1^ Department of Gastroenterological Surgery Hirosaki University Graduate School of Medicine Hirosaki Japan; ^2^ Department of Surgery, Tokyo Metropolitan Cancer and Infectious Diseases Center Komagome Hospital Tokyo Japan; ^3^ Department of Radiology Hirosaki University Graduate School of Medicine Hirosaki Japan; ^4^ Department of Surgery Yaizu City Hospital Yaizu Japan; ^5^ Department of Surgery National Defense Medical College Saitama Japan; ^6^ Department of Gastrointestinal Surgery Tokyo Medical and Dental University Tokyo Japan; ^7^ Department of Coloproctological Surgery Juntendo University Faculty of Medicine Tokyo Japan; ^8^ Department of Surgery Teikyo University Hospital Mizonokuchi Kawasaki Kanagawa Japan; ^9^ Division of Molecular and Diagnostic Pathology, Graduate School of Medical and Dental Sciences Niigata University Niigata Japan

**Keywords:** lateral lymph node dissection, lateral lymph node metastasis, MRI, neoadjuvant chemoradiotherapy, neoadjuvant chemotherapy, rectal cancer, upfront surgery

## Abstract

**Aim:**

The purpose was to clarify the oncological outcomes of rectal cancer (RC) with lateral lymph node metastasis (LLNM) on high‐resolution MRI (HRMRI), considering preoperative treatments.

**Methods:**

Two hundred and twelve patients, from 13 hospitals, diagnosed with RC with lateral lymph node dissection (LLND), between 2017 and 2019, were prospectively registered. LLNM was defined as a short‐axis size ≥5 mm. Ultimately, this study included 102 patients. Upfront surgery (Upfront), chemoradiotherapy (CRT), and neoadjuvant chemotherapy (NAC) were performed at each institution's discretion.

**Results:**

Sixty‐six (64.7%) had mesorectal fascia (MRF) involvement, 35 (34.3%) had extramural venous invasion, and 33 (32.4%) had bilateral LLNMs. A positive radial margin (RM1) was observed in nine patients (8.8%), and 35 (34.3%) had pathological LLNM (pLLNM). Overall, 3‐year relapse‐free survival (3yRFS) and local recurrence‐free survival (3yLRFS) were 69.6% and 92.9%. Upfront 3yRFS (*N* = 54), CRT (*N* = 23) and NAC (*N* = 25) constituted 62.9%, 82.6%, and 72.0%; 3yLRFS was 92.4%, 100%, and 88.0%. RM1 and pLLNM were significantly associated with LRFS (RM0 vs. RM1, 3yLRFS 96.7% vs. 50.0%; pLLNM negative vs. positive, 97.0% vs. 84.7%). 3yRFS Upfront non‐MRF (*N* = 21), post CRT non‐MRF (*N* = 15), and post NAC non‐MRF (*N* = 14) were 61.9%, 86.7%, and 100%; 3yLRFS was 90.2%, 100%, and 100%.

**Conclusions:**

Good local control of Upfront LLND for RC with LLNM was shown, but multidisciplinary treatments were required. CRT followed by surgery was preferable for RC with LLNM, but a radiation‐sparing strategy is promising for post NAC non‐MRF.

## Graphical abstract

Kaplan–Meier analyses of RFS (A) and LRFS (B) in non‐MRF groups. RFS, relapse‐free survival; LRFS, local recurrence‐free survival; MRF, mesorectal fascia; Upfront, upfront surgery; CRT, chemoradiotherapy; NAC, neoadjuvant chemotherapy.

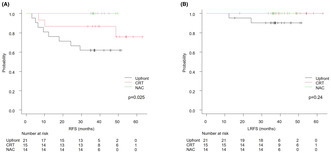

## 1 INTRODUCTION

Total mesorectal excision (TME) and lateral lymph node dissection (LLND) comprise the most highly recommended standard of care according to the Japanese guidelines for rectal cancer (RC) with lateral lymph node metastasis (LLNM).^1^ However, TME and LLND alone have resulted in high local and distant treatment failure in RC with pathological LLNM, so strategies to improve outcomes are required.^2^, ^3^, ^4^ On the contrary, good results in cases of TME and LLND for RC with LLNM conducted after preoperative chemoradiotherapy (CRT) have been reported at several centers in Japan.^5^, ^6^ This approach has been widely accepted not only in Japan but around the world, and is currently considered one of the most useful treatment strategies.^7^, ^8^ Recently, good results with TME and LLND after total neoadjuvant treatments (TNT) combining neoadjuvant radiotherapy and neoadjuvant chemotherapy (NAC) have also been reported.^9^, ^10^ Furthermore, TNT has been shown to produce a high rate of pCR. Reflecting this trend, the development of strategies to omit highly technical LLND is being considered.^9^, ^11^ Accordingly, treatment strategies for RC with LLNM are constantly evolving and transforming; but first, and foremost, the fact that the actual diagnoses for such cases have not been uniform makes it difficult to interpret the results of previous studies accurately.

This study was designed as a secondary analysis of an MRI study in which a Japanese multicenter prospective registry of RC patients, deemed eligible for LLND, were evaluated by high‐resolution MRI (HRMRI). The purpose of the study was to clarify the oncological outcomes of RC with LLNM on HRMRI at multiple centers in Japan, taking the detailed findings of HRMRI and preoperative treatments into consideration.

## 2 PATIENTS AND METHODS

### 2.1 Patients

A total of 212 patients who were diagnosed with rectal adenocarcinoma and had undergone TME and LLND between January 2017 and December 2019, as part of the Japanese Society for Cancer of the Colon and Rectum (JSCCR) MRI study group, comprising 13 affiliated referral hospitals, were prospectively registered. When the lower edge of an advanced tumor was below the peritoneal reflection (i.e., advanced lower RC), LLND was intended according to Japanese guidelines.^1^ LLND was also scheduled if the pretreatment MRI showed LLNM regardless of tumor location. Most of institutions with the policy of Upfront or NAC planned bilateral LLND. Almost all institutions with the policy of CRT planned selective LLND for the side of LLNMs on pretreatment MRI. Patients had all undergone MRI with a 3‐mm slice thickness on a high‐resolution T2‐weighted image before treatment. We excluded two patients due to lack of MRI records, one patient with concomitant prostate cancer, and an additional 96 patients without LLNM on MRI. Lymph node metastasis was defined as a short‐axis size ≥5 mm.^12^ Ultimately, 102 patients were included in this study after excluding 11 patients with distant metastases at registration. Preoperative therapy had been determined at the discretion of each institution. When the patient underwent preoperative therapy, MRI was also performed before surgery. This study was approved by the Ethics Committee of the University of Tokyo Hospital (No. 11406‐[5]), and written informed consent was obtained from each participant.

### 2.2 Evaluation of staging and treatment effects of high‐resolution MRI

The sizes of the lateral lymph nodes on MRI were measured by a single surgeon (KK) without clinical information. Tumor size, the distance from the anal verge, T stage, mesorectal fascia (MRF) involvement, extramural vascular invasion (EMVI), and the number of metastatic perirectal lymph nodes (PLNM) were all evaluated by one surgeon, TM, in consultation with a radiologist, HF, who had not been given any clinical information.^13^ When the patient underwent preoperative therapy, the same MRI results had been evaluated and an MRI tumor regression grade (mrTRG) assigned.^13^

### 2.3 Endpoint and statistical analyses

The categorical variables were compared using Fisher's exact test or the chi‐square test, and the continuous variables with the Mann–Whitney *U* test or Kruskal–Wallis test. Local recurrence‐free survival (LRFS) was calculated from the date of surgery to pelvic recurrence, relapse‐free survival (RFS) was calculated from the date of surgery to recurrence or death from any cause, and overall survival (OS) was calculated from the date of surgery to death from any cause, using the Kaplan–Meier method. Prognostic data were collected at 3 years after surgery. Univariate analysis was performed in RFS and LRFS using a log‐rank test. To investigate preoperative prognostic factors, candidate factors in univariate analysis (*p* < 0.10) were included in multivariate analysis using a Cox proportional hazards model. A two‐sided *p* < 0.05 was considered statistically significant, and all statistical analyses were performed with EZR (Saitama Medical Center, Jichi Medical University, Saitama, Japan), which is a graphical user interface for R (The R Foundation for Statistical Computing, Vienna, Austria).^14^

## 3 RESULTS

### 3.1 Patient characteristics

The median age was 62 years (29–82), and 61 (59.8%) of patients were male. The median body mass index (BMI) was 22.4 kg/m^2^ (16.0–32.9) and the median value of carcinoembryonic antigen (CEA) was 4.1 ng/mL (0.5–128.1). Upfront surgery without preoperative treatment (Upfront) was selected for 54 patients (53.0%), CRT was used on 23 patients (22.5%), and neoadjuvant chemotherapy (NAC) was utilized with 25 patients (24.5%). No patients underwent total neoadjuvant therapy. The average age of Upfront patients was significantly higher than those who received NAC (Table 1). There were no significant differences in gender, BMI, histology, or CEA between Upfront, CRT, or NAC. The chemotherapy regimens of CRT were as follows: 18 UFT/LV, 1 UFT/LV plus Irinotecan, 2 Capecitabin, 1 Irinotecan plus S‐1, and 1 S‐1. The radiotherapy of CRT was performed by the long‐course with a total dose of 45‐51Gy. The chemotherapy regimens of NAC were as follows: 16 FOLFOX/CAPOX/SOX, six FOLFOX/CAPOX/SOX plus Bevacizumab, and three FOLFOX plus Panitumumab. The intended courses of NAC were as follows: FOLFOX or FOLFOX plus Bevacizumab were six courses, CAPOX/SOX or CAPOX/SOX plus Bevacizumab 3–4 courses, FOLFOX plus Panitumumab 4–12 courses. The completion rate of NAC was 96%.

**TABLE 1:** The comparisons of clinicopathological and MRI findings between Upfront, CRT, and NAC.

Variables	Upfront	CRT	NAC	*p* Value
	*N* = 54	*N* = 23	*N* = 25	
Age^a^	66 (35–82)	63 (35–78)	57 (29–71)	<0.01
Gender (male), *n* (%)	30 (55.6)	15 (65.2)	16 (64.0)	0.69
Body mass index (kg/m^2^)^a^	22.1 (17.3–32.9)	21.3 (16.4–31.7)	22.9 (16.0–31.7)	0.53
Por/Muc, *n* (%)	5 (9.3)	3 (13.0)	0 (0)	0.20
CEA (ng/mL)^a^	4.0 (0.5–101.3)	5.5 (1.1–128.1)	4.1 (2.3–57.0)	0.23
Tumor size (mm)^a^	41 (20–120)	43 (30–91)	43 (22–100)	0.53
Distance from AV to tumor (mm)^a^	50 (0–95)	47 (0–101)	50 (0–90)	0.82
cT4, *n* (%)	5 (9.3)	5 (21.7)	7 (28.0)	0.075
MRF involvement, *n* (%)	33 (61.1)	17 (73.9)	16 (64.0)	0.60
EMVI positive, *n* (%)	14 (25.9)	8 (34.8)	13 (52.0)	0.082
cPLNM, *n* (%)	32 (59.3)	17 (73.9)	18 (72.0)	0.39
cLLNM, *n* (%)	54 (100)	23 (100)	25 (100)	
Number of PLNM^a^	2 (0–7)	1 (0–12)	2 (0–7)	0.70
Number of LLNM^a^	2 (1–7)	2 (1–5)	2 (1–7)	0.17
Maximum short‐axis size of LLNM (mm)^a^	6.3 (5.1–20.4)	8.4 (5.4–24.6)	7.3 (5.0–17.7)	<0.01
Bilateral LLNM, *n* (%)	17 (31.5)	5 (21.7)	11 (44.0)	0.25

Abbreviations: AV, anal verge; CEA, carcinoembryonic antigen; CRT, chemoradiotherapy; EMVI, extramural venous invasion; LLNM, lateral lymph node metastasis; MRF, mesorectal fascia; NAC, neoadjuvant chemotherapy; PLNM, perirectal lymph node metastasis; Por/Muc, poorly differentiated/mucinous adenocarcinoma; Upfront, upfront surgery.

^a^ Median (Range).

### 3.2 MRI evaluation

The median size of the tumor was 42 mm (20–120), and the median distance from the anal verge to the tumor was 50 mm (0–101). Seventeen (16.7%) were cT4, 66 (64.7%) had MRF involvement, 35 (34.3%) were EMVI positive, 67 (65.7%) were cPLNM, and the median number of PLNM was two (0–12). The median number of LLNM was two (1–7), and 33 (32.4%) were cases of bilateral LLNM. There were no clear differences in tumor size, distance from the anal verge, T stage, MRF, EMVI, cPLNM, number of PLNM/LLNM, or bilateral LLNMs between Upfront, CRT, or NAC (Table 1). There was a significantly shorter size of LLNM in Upfront (median, 6.3 mm) than in CRT (8.4 mm). MRI findings after preoperative treatment were compared between CRT and NAC (Table S1). There were no significant differences in tumor size, T stage, MRF, EMVI, tumor regression grade, cPLNM/LLNM, number of PLNM/LLNM, size of LLNM, or number of bilateral LLNMs between treatment with CRT and NAC.

### 3.3 Surgical and pathological outcomes

Eighty‐three (81.4%) cases were performed as laparoscopy or robot‐assisted surgery, while 61 (59.8%) were sphincter‐sparing. Bilateral LLND was performed in 74 cases (72.5%). There were no significant differences in terms of sphincter‐sparing surgery, laparoscopy or robot‐assisted procedures, blood loss, or operative time between Upfront, CRT, or NAC (Table 2). Bilateral LLND was significantly more often performed in Upfront (85.2%) and NAC (88.0%) cases as compared with CRT (26.1%). A positive radial margin (RM1) was observed in nine patients (8.8%), whereas 12 patients (11.8%) had pT4, 37 (36.3%) had pPLNM, and 35 (34.3%) had pLLNM. In terms of these findings, there were no significant differences between Upfront, CRT, or NAC. Fifty‐three patients (52.0%) underwent adjuvant chemotherapy, which was administered significantly more often in NAC cases (80.0%) than Upfront (42.6%) or CRT (43.5%). The adjuvant chemotherapy for patients with lymph node metastasis was performed in most of Upfront (75.0%), CRT (72.7%), and NAC (92.8%), respectively. Few patients without lymph node metastasis underwent adjuvant chemotherapy in Upfront (7.7%) and CRT (16.7%), but more than half of patients had adjuvant chemotherapy in NAC (63.6%).

**TABLE 2:** The comparisons of surgical and pathological outcomes between Upfront, CRT, and NAC.

Variables	Upfront	CRT	NAC	*p* Value
	*N* = 54	*N* = 23	*N* = 25	
SPS	33 (61.1)	14 (60.9)	14 (56.0)	0.92
Bilateral LLND, *n* (%)	46 (85.2)	6 (26.1)	22 (88.0)	<0.01
Laparoscopy/Robot, *n* (%)	41 (75.9)	19 (82.6)	23 (92.0)	0.24
Robot, *n* (%)	16 (29.6)	5 (21.7)	2 (8.0)	0.090
Blood loss (mL)^a^	130 (0–1714)	150 (30–1540)	130 (0–1260)	0.16
Operative time (mm)^a^	466 (242–855)	474 (274–808)	522 (297–765)	0.082
pT4, *n* (%)	6 (11.1)	4 (17.4)	2 (8.0)	0.60
RM1, *n* (%)	3 (5.6)	3 (13.0)	3 (12.0)	0.41
Pathologic response				
0		0	1 (4.0)	
1a		4 (17.4)	10 (40.0)	
1b		10 (43.5)	6 (24.0)	
2		9 (39.1)	7 (28.0)	
3		0	1 (4.0)	
pPLNM, *n* (%)	21 (38.9)	7 (30.4)	9 (36.0)	0.82
pLLNM, *n* (%)	18 (33.3)	8 (34.8)	9 (36.0)	0.96
pLNM, *n* (%)	28 (51.9)	11 (47.8)	14 (56.0)	0.83
Adjuvant chemotherapy, *n* (%)	23 (42.6)	10 (43.5)	20 (80.0)	<0.01

Abbreviations: CRT, chemoradiotherapy; LLND, lateral lymph node dissection; LLNM, lateral lymph node metastasis; LNM, lymph node metastasis; NAC, neoadjuvant chemotherapy; PLNM, perirectal lymph node metastasis; RM, radial margin; SPS, sphincter function‐preserving surgery; Upfront, upfront surgery.

^a^ Median (Range).

### 3.4 Oncological outcomes

**FIGURE 1:**
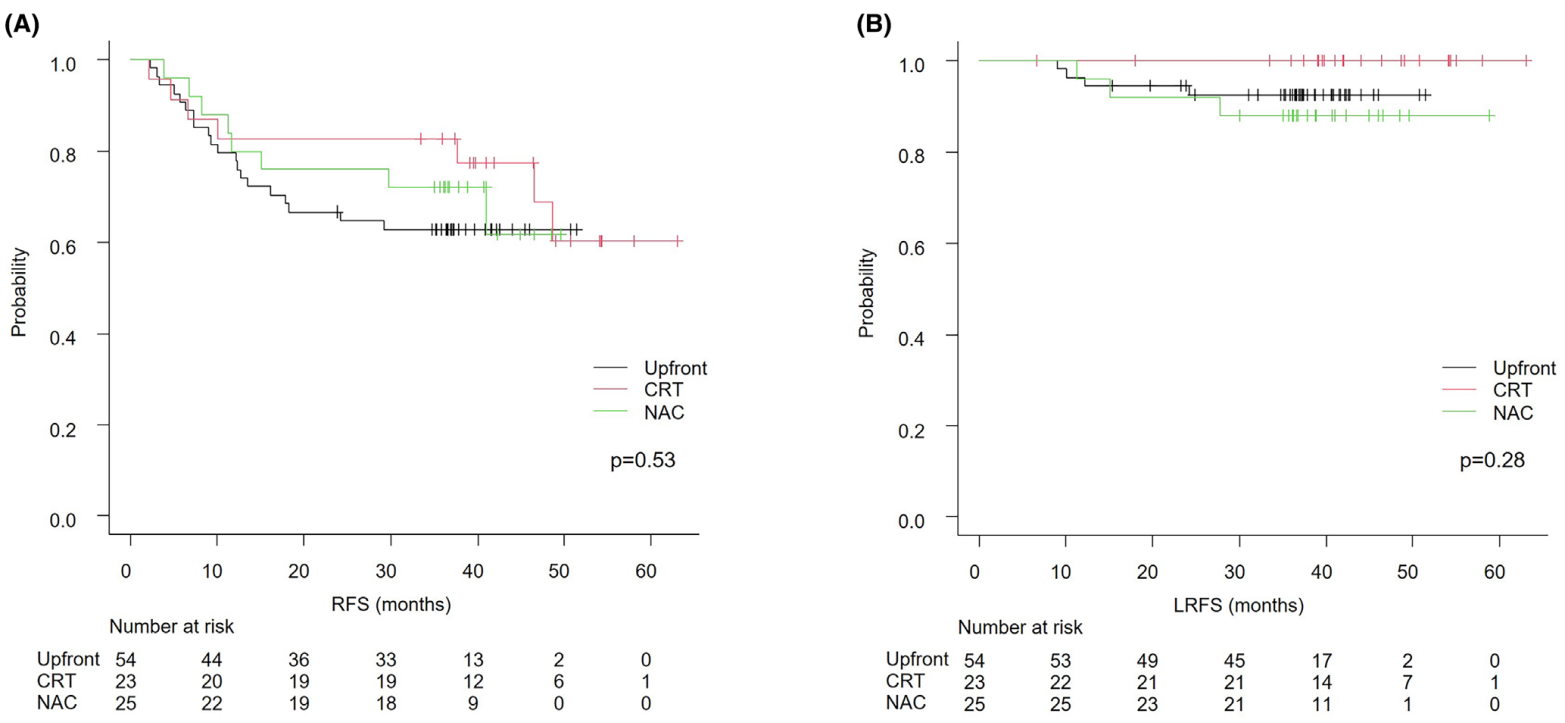
Kaplan–Meier analyses of RFS (A) and LRFS (B). CRT, chemoradiotherapy; LRFS, local recurrence‐free survival; NAC, neoadjuvant chemotherapy; RFS, relapse‐free survival; Upfront, upfront surgery.

The median observation period was 38 (6–63) months. Overall, 3‐year OS, RFS (3yRFS), and LRFS (3yLRFS) were 91.9%, 69.6%, and 92.9%, respectively. The 3yOS rates for Upfront, CRT, and NAC were 90.3%, 95.7%, and 92.0% (*p* = 0.21), respectively. The 3yRFS were 62.9%, 82.6%, and 72.0% (*p* = 0.53), respectively (Figure 1). The 3yLRFS was 92.4%, 100%, and 88.0% (*p* = 0.28), respectively. In multivariate analysis, poorly differentiated or mucinous adenocarcinoma was independently correlated with RFS (Table 3). Post‐MRF status showed poor RFS, but it was not independently significant (hazard ratio 1.75, *p* = 0.18). Adjuvant chemotherapy was not significantly associated with RFS (non‐adjuvant vs. adjuvant, 3yRFS 75.5% vs. 64.2%, *p* = 0.39). No independently associated preoperative factors were found in terms of LRFS (Table S2). Of the pathological findings, RM and pLLNM were significantly associated with LRFS (RM0 vs. RM1, 3yLRFS 96.7% vs. 50.0%, *p* < 0.01; pLLNM negative vs. positive, 97.0% vs. 84.7%, *p* = 0.026).

**TABLE 3:** Univariate and multivariate analysis in preoperative factors for relapse‐free survival.

Variables	*N*	Univariate		Multivariate	*p* Value
		3yRFS (%)	*p* Value	HR (95% CI)	
Age			0.96		
<60	46	67.4			
≥60	56	71.4			
Gender			0.77		
Female	41	68.3			
Male	61	70.4			
Histology			0.032		0.018
Pap/Well/Mod	94	71.2		1.00	
Por/Muc	8	50.0		3.47 (1.23–9.83)	
CEA (ng/mL)			0.022		0.078
<5.0	58	79.3		1.00	
≥5.0	44	56.6		1.89 (0.92–3.87)	
Tumor size (mm)			0.15		
<50	71	73.2			
≥50	31	61.1			
Distance from AV (mm)			0.13		
<50	49	75.5			
≥50	53	64.2			
Preoperative treatment			0.30		
Yes	48	77.1			
No	54	62.9			
CRT			0.34		
Yes	23	82.6			
No	79	65.8			
Adjuvant chemotherapy			0.39		
Yes	53	64.2			
No	49	75.5			
Robot			0.46		
Yes	23	64.9			
No	79	70.9			
cT stage			0.28		
cT≤3	85	71.7			
cT4	17	58.8			
ycT stage^a^			0.17		
ycT≤3	88	71.5			
ycT4	14	57.1			
pre‐PLNM			0.034		0.82
Negative	35	85.7		1.00	
Positive	67	61.1		1.14 (0.35–3.70)	
post‐PLNM^a^			<0.01		0.19
Negative	56	83.9		1.00	
Positive	46	52.0		2.06 (0.68–6.23)	
pre‐LLNM			0.60		
Unilateral	69	71.0			
Bilateral	33	66.4			
post‐LLNM^a^			0.039		0.33
Negative	16	93.8		1.000	
Positive	86	65.1		2.11 (0.45–9.84)	
pre‐MRF status			0.27		
Negative	36	75.0			
Positive	66	66.6			
post‐MRF status^a^			<0.01		0.18
Negative	50	80.0		1.000	
Positive	52	59.6		1.75 (0.77–3.98)	
pre‐EMVI status			0.20		
Negative	67	71.6			
Positive	35	65.7			
post‐EMVI status^a^			0.030		0.14
Negative	79	73.4		1.00	
Positive	23	56.5		1.76 (0.82–3.78)	

Abbreviations: 3yRFS, 3‐year relapse‐free survival; AV, anal verge; CEA, carcinoembryonic antigen; CI, confidence interval; CRT, chemoradiotherapy; HR, hazard ratio; LLNM, lateral lymph node metastasis; MRF, mesorectal fascia, EMVI, extramural venous invasion; Pap/Well/Mod, papillary/well/moderately differentiated tubular adenocarcinoma; PLNM, perirectal lymph node metastasis; Por/Muc, poorly differentiated/mucinous adenocarcinoma; post‐, posttreatment; pre‐, pretreatment; TRG, tumor regression grade.

^a^ Post‐findings of Upfront were substituted by pre‐findings.

### 3.5 Upfront non‐MRF, post CRT non‐MRF, or post NAC non‐MRF

**FIGURE 2:**
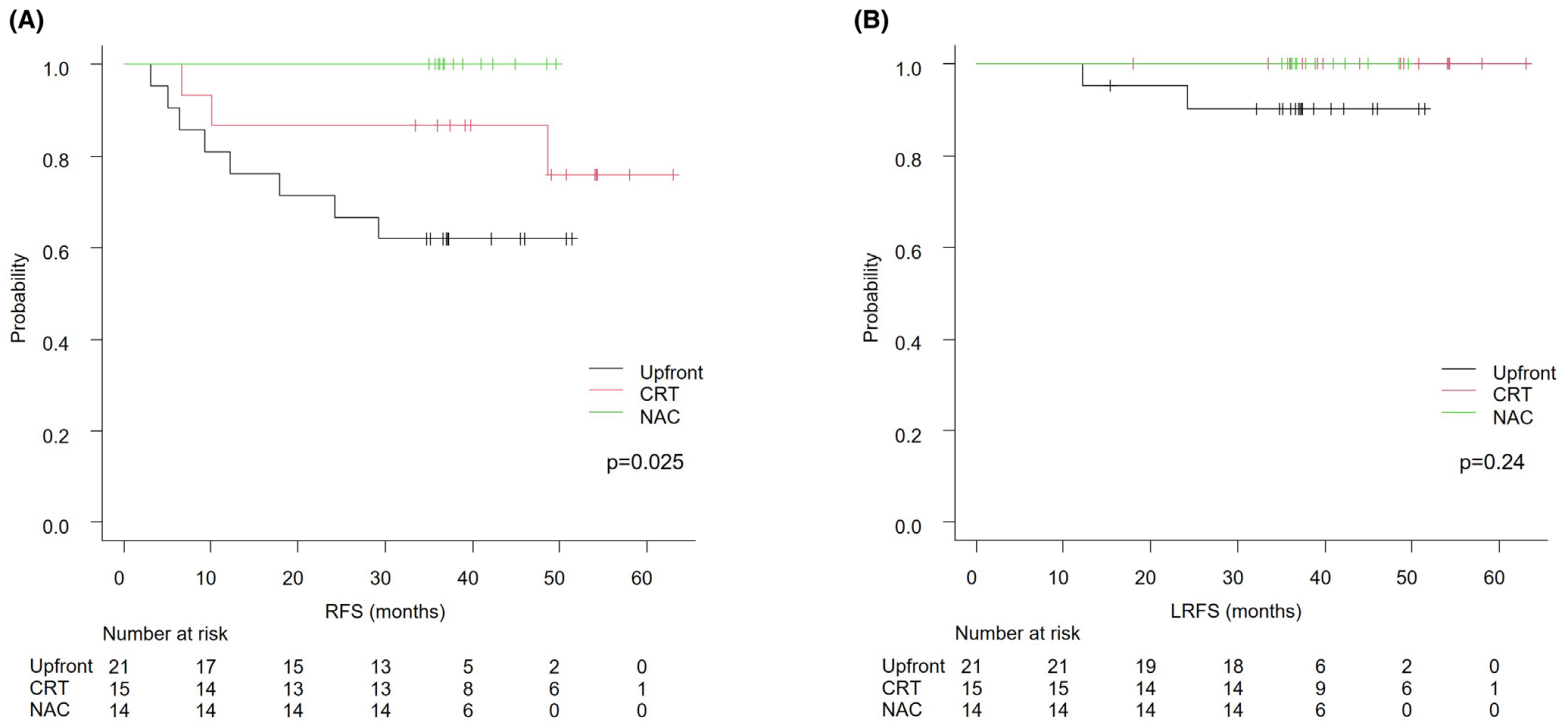
Kaplan–Meier analyses of RFS (A) and LRFS (B) in non‐MRF groups. CRT, chemoradiotherapy; LRFS, local recurrence‐free survival; MRF, mesorectal fascia; NAC, neoadjuvant chemotherapy; RFS, relapse‐free survival; Upfront, upfront surgery.

Upfront non‐MRF comprised 21 patients, post CRT non‐MRF consisted of 15 patients, and post NAC non‐MRF included 14 patients. MRF involvement was observed in the pretreatment‐MRI of nine (60.0%) of the post CRT non‐MRF patients and five (35.7%) of the post NAC non‐MRF cases. The average age of Upfront patients was significantly higher than those who received NAC (Table 4). There were no significant differences in gender, BMI, histology, or CEA between Upfront, CRT, or NAC. In terms of pretreatment‐MRI, there were no clear differences in tumor size, distance from the anal verge, T stage, EMVI, cPLNM, number of PLNM/LLNM, or bilateral LLNM between Upfront, CRT, or NAC. There was a significantly shorter size of LLNM with Upfront (median, 6.1 mm) than with CRT (8.2 mm). RM1 was not observed in any patients, and there were no clear differences in pT4, pPLNM, and pLLNM between Upfront, CRT, or NAC. Adjuvant chemotherapy significantly was administered more often to those who had received NAC (85.7%) than those treated with Upfront (33.3%) or CRT (40.0%). The 3yRFS for Upfront non‐MRF, post CRT non‐MRF, and post NAC non‐MRF were 61.9%, 86.7%, and 100% (*p* = 0.025), respectively; the 3yLRFS was 90.2%, 100%, and 100% (*p* = 0.24; Figure 2). The 3yRFS for Upfront MRF (*N* = 33), post CRT MRF (*N* = 8), and post NAC MRF (*N* = 11) were 63.6%, 75.0%, and 36.4% (*p* = 0.14), respectively; the 3yLRFS was 93.9%, 100% and 72.7% (*p* = 0.10; Figure S1). There was a significantly higher rate of cT4 with CRT (50.0%) and NAC (54.5%) than with Upfront (15.2%; Table S3).

**TABLE 4:** The comparisons of clinicopathological and MRI findings between Upfront non‐MRF, post CRT non‐MRF, and post NAC non‐MRF.

Variables	Upfront	CRT	NAC	*p* Value
	*N* = 21	*N* = 15	*N* = 14	
Age^a^	63 (51–79)	60 (35–76)	57 (35–70)	0.038
Gender (male), *n* (%)	13 (61.9)	11 (73.3)	8 (57.1)	0.70
Body mass index (kg/m^2^)^a^	21.8 (19.3–28.4)	23.0 (17.1–31.7)	22.5 (16.0–29.4)	0.93
Por/Muc, *n* (%)	2 (9.5)	1 (6.7)	0 (0)	0.77
CEA (ng/mL)^a^	3.1 (0.5–39.1)	4.9 (1.1–54.4)	3.8 (2.3–17.6)	0.13
Tumor size (mm)^a^	34 (20–71)	36 (30–61)	40 (22–50)	0.46
Distance from AV to tumor (mm)^a^	55 (27–91)	44 (20–101)	60 (15–90)	0.53
cT4, *n* (%)	0 (0)	1 (6.7)	1 (7.1)	0.50
MRF involvement, *n* (%)	0 (0)	9 (60.0)	5 (35.7)	<0.01
EMVI positive, *n* (%)	3 (14.3)	5 (33.3)	7 (50.0)	0.078
cPLNM, *n* (%)	10 (47.6)	11 (73.3)	8 (57.1)	0.31
cLLNM, *n* (%)	21 (100)	15 (100)	14 (100)	
Number of PLNM^a^	0 (0–7)	1 (0–9)	2 (0–7)	0.54
Number of LLNM^a^	2 (1–4)	2 (1–5)	2 (1–5)	0.79
Maximum short‐axis size of LLNM (mm)^a^	6.1 (5.1–12.2)	8.2 (5.4–24.6)	7.2 (5.2–16.3)	<0.01
Bilateral LLNM, *n* (%)	7 (33.3)	3 (20.0)	6 (42.9)	0.43
pT4, *n* (%)	0 (0)	1 (6.7)	0 (0)	0.58
RM1, *n* (%)	0 (0)	0 (0)	0 (0)	
pPLNM, *n* (%)	8 (38.1)	3 (20.0)	4 (28.6)	0.49
pLLNM, *n* (%)	6 (28.6)	5 (33.3)	3 (21.4)	0.85
Adjuvant chemotherapy, *n* (%)	7 (33.3)	6 (40.0)	12 (85.7)	<0.01

Abbreviations: AV, anal verge; CEA, carcinoembryonic antigen; CRT, chemoradiotherapy; EMVI, extramural venous invasion; LLNM, lateral lymph node metastasis; MRF, mesorectal fascia; NAC, neoadjuvant chemotherapy; PLNM, perirectal lymph node metastasis; Por/Muc, poorly differentiated/mucinous adenocarcinoma; RM, radial margin; Upfront, upfront surgery.

^a^ Median (Range).

## 4 DISCUSSION

This is the first multicenter prospective study of LLND for RC with LLNM assessed by HRMRI. The novelty of this study is that it includes cases treated with the multiple modalities of Upfront, CRT, and NAC, because it was registered during a time when preoperative CRT was not specified as a treatment standard in the Japanese guidelines.

LLND for RC with LLNM is strongly recommended in the Japanese guidelines and that importance is also recognized around the globe.^1^, ^8^ Preoperative CRT for RC with a risk of local recurrence is a standard treatment around the world, and, more recently, in Japan as well.^1^, ^15^, ^16^ The results of several randomized control trials to date indicate that cStage II‐III RC carries a risk for local recurrence, but there are differences among centers in their criteria for introducing CRT.^17^, ^18^ The selective use of CRT has been provided based on the findings from HRMRI studies which have clarified the risks of recurrence beyond TNM classification.^13^, ^19^ Important risk factors for recurrence shown in those studies include MRF involvement and EMVI.^20^, ^21^ These factors are now widely accepted and incorporated into the ESMO guidelines as important indicators for risk stratification, upon which treatment strategy is decided.^15^

The diagnosis of LLNM has been reported in terms of short‐axis size, long‐axis size, and morphology, but there is still no uniformity in the literature.^5^, ^6^, ^7^, ^8^, ^12^, ^22^ In our study, we adopted a short‐axis size ≥5 mm, which proved to be a good indicator of LLNM and LLND, as attested to by the lymph node committee of the JSCCR and by the Japan Society of Laparoscopic Colorectal Surgery.^12^, ^23^ In our study, the positive rate of metastasis in a short‐axis size ≥5 mm on HRMRI was 33.3% in Upfront cases, and 34%–36% after CRT or NAC. Although not diagnosed using HRMRI, the positive rate of metastasis with a short‐axis size ≥5 mm was 21.4%–47.7% with Upfront.^12^, ^24^ The MRI committee of JSCCR are including this cohort in their work on the development of sensitivity‐oriented diagnostic criteria for the diagnosis of LLNM, so these criteria will be validated in the future.^25^

Long‐term outcomes of LLND for RC with LLNM defined as MRI short‐axis size ≥5 mm has shown a 5‐year RFS of 69.4% in a cohort that included 28.1% with preoperative treatments (18.9% CRT).^23^ The 3yRFS and 3‐year local recurrence rate of post‐CRT LLND for RC with LLNM defined as MRI short‐axis size ≥5 mm were reported to be 77.1% and 5.3%, respectively.^7^ In our study, 3yRFS and 3yLRFS were 62.9% and 92.4% in Upfront, and, in CRT, 82.6% and 100%, respectively. Considering these results, Upfront LLND had good local control but poor outcomes for distant metastasis. Such outcomes may be influenced by the low rate of adjuvant therapy (42.6%) after Upfront LLND. RC with LLNM on HRMRI suggests the necessity of multidisciplinary treatments even if Upfront LLND is expected to be a curative resection.

On the other hand, NAC was 72.0% in terms of the 3‐year RFS and was 88.0% for the 3‐year LRFS in our study. Although NAC showed good long‐term results equivalent to CRT in RC without LLNM, it was considered insufficient as a preoperative treatment for RC with LLNM.^26^, ^27^ However, as shown in our study, LLNM cases are mainly low lying with a distance from the anal verge to the tumor of about 5 cm, where bowel dysfunction associated with TME is inevitable and worsening of bowel function due to radiation is a concern.^28^ In addition, RC has become more common at a younger age, in recent years, raising concerns about radiation‐related infertility, late bone damage, and secondary carcinogenesis.^29^, ^30^, ^31^ Therefore, the establishment of a non‐radiotherapy strategy that takes these considerations into account is also warranted. In this study, RM1 was the most responsible for local recurrence in RC with LLNM. This pathological finding can be predicted preoperatively using HRMRI based on MRF involvement.^32^ Therefore, if LLNM is noted but MRF is negative, a high cure rate might be expected with TME and LLND alone. Likewise, in our study, RM1 was not observed in any cases of upfront non‐MRF or post CRT/NAC‐non MRF. However, 3yRFS and 3yLRFS for upfront non‐MRF were 61.5% and 90.2%, respectively, which were not the same as post CRT‐non MRF (86.7% and 100%) and post NAC‐non MRF (100% and 100%). These results suggest that NAC without radiation should be considered as one viable multidisciplinary treatment in addition to LLND for RC with LLNM when non‐MRF conditions are observed.

In recent years, TNT has shown promise as a most effective preoperative treatment strategy.^33^, ^34^ However, the high local recurrence rates after TNT, which were reported in the RAPIDO trial compared with CRT in 5‐year follow‐up, has prompted a rethinking of better multidisciplinary treatment strategies.^35^ Difficulty in surgery after prolonged waiting time after radiotherapy has been thought to be a factor in local recurrence after TNT. In cases with LLNM, not only the difficulty of TME but also the difficulty of LLND should be considered. LLND after TNT for RC with LLNM has also been reported with good results, but this is based on a single‐center, retrospective study, for which interpretation remains limited.^9^, ^10^ Furthermore, considering the very good results with CRT followed by LLND in our study, the most appropriate preoperative treatment for RC with LLNM merits further investigation.

LLND, on the other hand, is a technique that requires advanced skills and is associated with increased postoperative complications. Actually, although they were not statistically different, the longer operative time was shown in NAC than Upfront. Also, there was similar operative time between CRT and Upfront despite bilateral LLND being less common in CRT. These data indicated CRT and NAC might increase the difficulty of TME and LLND due to the edema and fibrosis, so non‐LLND strategies are also warranted. Prediction of the disappearance of LLNM after preoperative treatment is important, and a short‐axis size of 4 mm or less has been reported as one indicator.^11^ However, this report is also the result of a retrospective study, and an appropriately designed prospective study of non‐LLND strategies for RC with LLNM that completely disappear after preoperative treatment is required.

Although this is a multicenter prospective study, there are several limitations. Indication criteria for preoperative and postoperative adjuvant therapy are left to the institution, and treatment selection bias may exist. In addition, there is no unified regimen for CRT, NAC, or postoperative adjuvant therapy. The genetic status such as RAS, BRAF, and MSI was not investigated in this study. HRMRIs are centrally evaluated, but they are not done by uniformly experienced radiologists. More cases are required to investigate preoperative therapy at a sufficient level of statistical validity and reproducibility to achieve significance.

## 5 CONCLUSIONS

Prospective multicenter oncological outcomes were clarified for LLND in RC with LLNM diagnosed on HRMRI. Good local control of Upfront LLND for RC with LLNM on HRMRI was shown, but multidisciplinary treatments were considered to be required. It was suggested that CRT followed by surgery may be preferable for RC with LLNM on HRMRI, but a radiation‐sparing strategy can be expected for post NAC non‐MRF cases.

## AUTHOR CONTRIBUTIONS

TM and KK contributed to the conception and design of the study. TM, KK, HF, SK, HU, YK, KS, and HK performed the data acquisition. TM, KK, and HF were in charge of analysis and/or interpretation of data for the study. TM drafted the manuscript. KH and YA contributed to the review and/or critical revision of the article.

## FUNDING INFORMATION

This study was funded by JSCCR.

## CONFLICT OF INTEREST STATEMENT

Authors HU, YK, and KH are editorial members of *Annals of Gastroenterological Surgery*.

## ETHICS STATEMENT

Approval of the research protocol by an Institutional Reviewer Board: This study was conducted with the Ethics Committee of the University of Tokyo Hospital (11406‐[5]).

Informed Consent: Written informed consent was obtained from each patient before enrollment.

Registry and the Registration No. of the study/trial: N/A.

Animal Studies: N/A.

## Supporting information


Figure S1.

Table S1.
Table S2.
