# Supplementary material for: Upfront surgery, neoadjuvant chemoradiotherapy, or neoadjuvant chemotherapy for rectal cancer with lateral lymph node metastasis: A multicenter MRI and lateral lymph node dissection study
Source: Ann Gastroenterol Surg. 2024 Oct 16;9(2):309–18. doi: 10.1002/ags3.12873 (PMC11877350; doi:10.1002/ags3.12873)
Supplement: Supplementary file 1 — Figure S1. [file AGS3-9-309-s002.pdf]

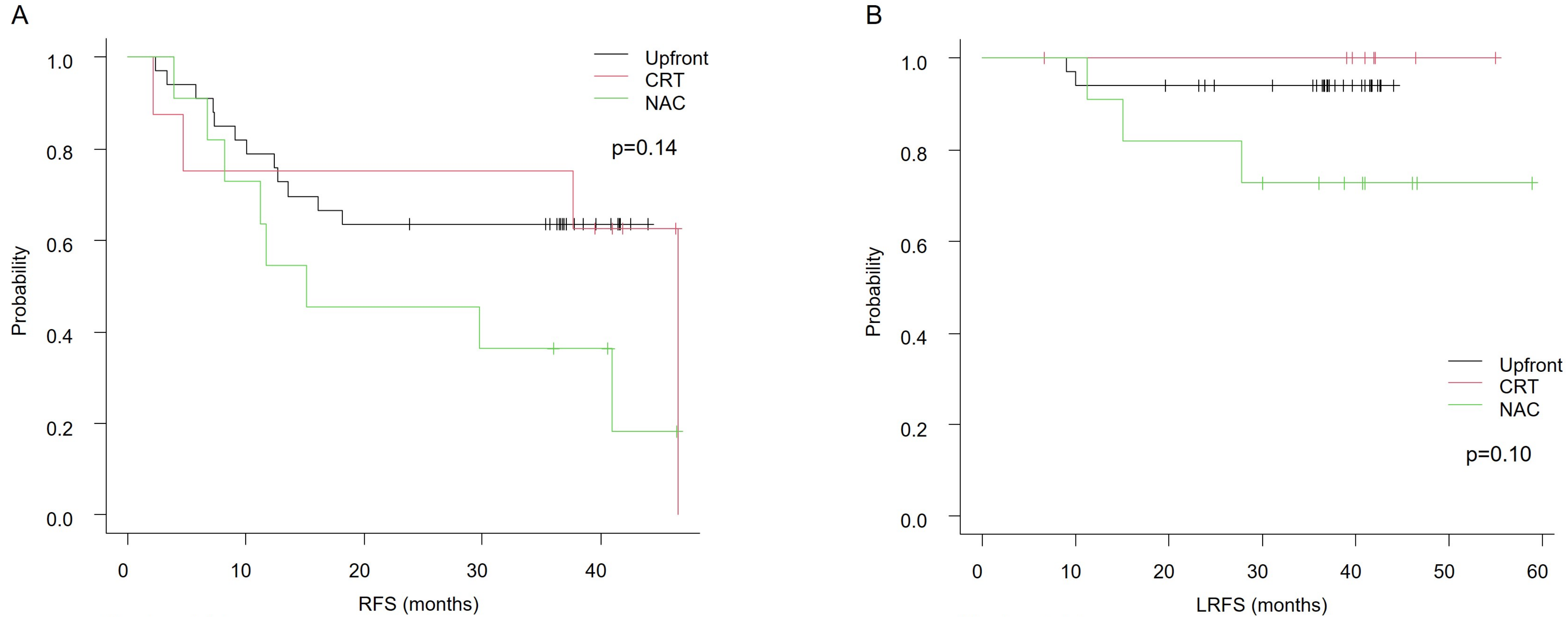

Number at risk (A)

	0	10	20	30	40
Upfront	33	27	21	20	8
CRT	8	6	6	6	4
NAC	11	8	5	4	3

Number at risk (B)

	0	10	20	30	40	50	60
Upfront	33	32	30	27	11	0	0
CRT	8	7	7	7	5	1	0
NAC	11	11	9	7	5	1	0

Sup. Fig. 1. Kaplan-Meier analyses of RFS (A) and LRFS (B) in MRF groups. RFS, relapse-free survival; LRFS, local recurrence-free survival; MRF, mesorectal fascia; Upfront, upfront surgery; CRT, chemoradiotherapy; NAC, neoadjuvant chemotherapy.
