# Supplementary material for: Upfront surgery, neoadjuvant chemoradiotherapy, or neoadjuvant chemotherapy for rectal cancer with lateral lymph node metastasis: A multicenter MRI and lateral lymph node dissection study
Source: Ann Gastroenterol Surg. 2024 Oct 16;9(2):309–18. doi: 10.1002/ags3.12873 (PMC11877350; doi:10.1002/ags3.12873)
Supplement: Supplementary file 2 — Table S1. Table S2. [file AGS3-9-309-s001.docx]

Sup Table 1. The comparisons of MRI findings after preoperative therapy between CRT and NAC

| Variables | CRT | NAC | *P* value |
| --- | --- | --- | --- |
|  | N=23 | N=25 |  |
| Tumor size (mm) † | 20 (0-82) | 28 (0-72) | 0.13 |
| cT4, n (%) | 4 (17.4) | 5 (20.0) | 1.00 |
| MRF involvement, n (%) | 8 (34.8) | 11 (44.0) | 0.56 |
| EMVI positive, n (%) | 3 (13.0) | 6 (24.0) | 0.46 |
| Tumor regression grade 1-2, n (%) | 9 (39.1) | 5 (20.0) | 0.20 |
| cPLNM, n (%) | 5 (21.7) | 9 (36.0) | 0.34 |
| cLLNM, n (%) | 16 (69.6) | 16 (64.0) | 0.76 |
| Number of PLNM † | 0 (0-2) | 0 (0-6) | 0.14 |
| Number of LLNM † | 1 (0-2) | 1 (0-6) | 0.46 |
| Maximum short-axis size of LLNM (mm) † | 5.6 (3.4-16.6) | 5.6 (4.1-18.5) | 0.93 |
| Bilateral LLNM, n (%) | 2 (8.7) | 8 (32.0) | 0.075 |

† Median (Range)

CRT, chemoradiotherapy; NAC, neoadjuvant chemotherapy; AV, anal verge; MRF, mesorectal fascia; EMVI, extramural venous invasion; PLNM, perirectal lymph node metastasis; LLNM, lateral lymph node metastasis.

Sup Table 2. Univariate and multivariate analysis in preoperative factors for local recurrence-free survival

|  |  | Univariate | | Multivariate |  |
| --- | --- | --- | --- | --- | --- |
| Variables | N | 3yLRFS (%) | *P* value | HR (95% CI) | *P* value |
| Age |  |  | 0.91 |  |  |
| < 60 | 46 | 93.2 |  |  |  |
| ≥ 60 | 56 | 92.7 |  |  |  |
| Gender |  |  | 0.53 |  |  |
| Female | 41 | 95.1 |  |  |  |
| Male | 61 | 91.6 |  |  |  |
| Histology |  |  | 0.010 |  | 0.10 |
| Pap/Well/Mod | 94 | 94.6 |  | 1.00 |  |
| Por/Muc | 8 | 68.6 |  | 3.97 (0.75-20.8) |  |
| CEA (ng/mL) |  |  | 0.42 |  |  |
| <5.0 | 58 | 94.7 |  |  |  |
| ≥5.0 | 44 | 90.7 |  |  |  |
| Tumor size (mm) |  |  | 0.43 |  |  |
| <50 | 71 | 94.3 |  |  |  |
| ≥50 | 31 | 89.6 |  |  |  |
| Distance from AV (mm) |  |  | 0.78 |  |  |
| <50 | 49 | 93.7 |  |  |  |
| ≥50 | 53 | 92.3 |  |  |  |
| Preoperative treatment |  |  | 0.80 |  |  |
| Yes | 48 | 93.6 |  |  |  |
| No | 54 | 92.4 |  |  |  |
| CRT |  |  | 0.15 |  |  |
| Yes | 23 | 100 |  |  |  |
| No | 79 | 91.0 |  |  |  |
| Adjuvant chemotherapy |  |  | < 0.01 | NA | 0.99 |
| Yes | 53 | 86.5 |  |  |  |
| No | 49 | 100 |  |  |  |
| Robot |  |  |  |  |  |
| Yes | 23 | 95.6 | 0.59 |  |  |
| No | 79 | 92.2 |  |  |  |
| cT stage |  |  | 0.064 | NA | 0.99 |
| cT≤3 | 85 | 95.2 |  |  |  |
| cT4 | 17 | 82.4 |  |  |  |
| ycT stage* |  |  | 0.024 | NA | 0.99 |
| ycT≤3 | 88 | 95.3 |  |  |  |
| ycT4 | 14 | 78.6 |  |  |  |
| pre-PLNM |  |  | 0.72 |  |  |
| negative | 35 | 94.2 |  |  |  |
| positive | 67 | 92.3 |  |  |  |
| post-PLNM* |  |  | 0.46 |  |  |
| negative | 56 | 94.5 |  |  |  |
| positive | 46 | 91.1 |  |  |  |
| pre-LLNM |  |  | 0.81 |  |  |
| unilateral | 69 | 92.6 |  |  |  |
| bilateral | 33 | 93.6 |  |  |  |
| post-LLNM* |  |  | 0.23 |  |  |
| negative | 16 | 100 |  |  |  |
| positive | 86 | 91.6 |  |  |  |
| pre-MRF status |  |  | 0.66 |  |  |
| negative | 36 | 94.4 |  |  |  |
| positive | 66 | 92.2 |  |  |  |
| post-MRF status* |  |  | 0.24 |  |  |
| negative | 50 | 95.9 |  |  |  |
| positive | 52 | 90.0 |  |  |  |
| pre-EMVI status |  |  | 0.80 |  |  |
| negative | 67 | 92.5 |  |  |  |
| positive | 35 | 94.1 |  |  |  |
| post-EMVI status* |  |  | 0.65 |  |  |
| negative | 79 | 93.5 |  |  |  |
| positive | 23 | 91.3 |  |  |  |

3yLRFS, 3-year local recurrence-free survival; HR, hazard ratio; CI, confidence interval; Pap/Well/Mod, papillary/well/moderately differentiated tubular adenocarcinoma; Por/Muc, poorly differentiated/mucinous adenocarcinoma; CEA, carcinoembryonic antigen; AV, anal verge; CRT, chemoradiotherapty; pre-, pretreatment; PLNM, perirectal lymph node metastasis; LLNM, lateral lymph node metastasis; post-, posttreatment; TRG, tumor regression grade; MRF, mesorectal fascia, EMVI, extramural venous invasion; NA, not available. *Post-findings of Upfront were substituted by pre-findings.

Sup Table 3. The comparisons of clinicopathological and MRI findings between Upfront MRF, post CRT MRF and post NAC MRF

| Variables | Upfront | CRT | NAC | *P* value |
| --- | --- | --- | --- | --- |
|  | N=33 | N=8 | N=11 |  |
| Age† | 66 (49-79) | 68 (35-76) | 57 (35-70) | 0.065 |
| Gender (male), n (%) | 17 (51.5) | 4 (50.0) | 8 (72.7) | 0.48 |
| Body mass index (kg/m^2^) † | 22.4  (17.3-32.9) | 20.4  (16.4-29.0) | 22.9  (18.1-31.7) | 0.18 |
| Por/Muc, n (%) | 3 (9.1) | 3 (25.0) | 0 (0) | 0.15 |
| CEA (ng/ml) † | 5.0 (0.8-101.3) | 9.4 (1.8-128.1) | 5.9 (2.3-57.0) | 0.32 |
| Tumor size (mm) † | 43 (25-120) | 54 (39-91) | 54 (35-100) | 0.057 |
| Distance from AV to tumor (mm) † | 49 (0-95) | 51 (0-73) | 41 (0-87) | 0.89 |
| cT4, n (%) | 5 (15.2) | 4 (50.0) | 6 (54.5) | 0.014 |
| MRF involvement, n (%) | 33 (100) | 8 (100) | 11 (100) |  |
| EMVI positive, n (%) | 11 (33.3) | 3 (37.5) | 6 (54.5) | 0.49 |
| cPLNM, n (%) | 22 (66.7) | 6 (75.0) | 10 (90.9) | 0.38 |
| cLLNM, n (%) | 33 (100) | 8 (100) | 11 (100) |  |
| Number of PLNM † | 2 (0-7) | 3 (0-12) | 2 (0-4) | 0.72 |
| Number of LLNM † | 1 (1-7) | 2 (1-2) | 2 (1-7) | 0.66 |
| Maximum short-axis size of LLNM (mm) † | 6.3 (5.1-20.4) | 10.2 (5.7-17.5) | 9.5 (5.0-17.7) | 0.087 |
| Bilateral LLNM, n (%) | 10 (30.3) | 2 (25.0) | 5 (45.5) | 0.68 |
| pT4, n (%) | 6 (18.2) | 3 (37.5) | 2 (18.2) | 0.54 |
| RM1, n (%) | 3 (9.1) | 3 (37.5) | 3 (27.3) | 0.077 |
| pPLNM, n (%) | 15 (45.5) | 4 (50.0) | 5 (50.0) | 1.00 |
| pLLNM, n (%) | 12 (36.4) | 3 (37.5) | 6 (54.5) | 0.51 |
| Adjuvant chemotherapy, n (%) | 16 (48.5) | 4 (50.0) | 8 (72.7) | 0.37 |

† Median (Range)

Upfront, upfront surgery; CRT, chemoradiotherapy; NAC, neoadjuvant chemotherapy; MRF, mesorectal fascia; Por/Muc, poorly differentiated/mucinous adenocarcinoma; CEA, carcinoembryonic antigen; AV, anal verge; EMVI, extramural venous invasion; PLNM, perirectal lymph node metastasis; LLNM, lateral lymph node metastasis; RM radial margin.
